# Staff perspectives on the feasibility of a clinical pathway for anxiety and depression in cancer care, and mid-implementation adaptations

**DOI:** 10.1186/s12913-022-07532-2

**Published:** 2022-02-14

**Authors:** Phyllis Butow, Heather L. Shepherd, Jessica Cuddy, Nicole Rankin, Marnie Harris, Sharon He, Peter Grimison, Afaf Girgis, Mona Faris, The ADAPT Program Group, Philip Beale, Phyllis Butow, Josephine Clayton, Jessica Cuddy, Fiona Davies, Haryana Dhillon, Mona Faris, Liesbeth Geerligs, Afaf Girgis, Peter Grimison, Tom Hack, Marnie Harris, Sharon He, Brian Kelly, Patrick Kelly, Laura Kirsten, Toni Lindsay, Melanie Lovell, Tim Luckett, Lindy Masya, Michael Murphy, Jill Newby, Don Piro, Nicole Rankin, Joanne Shaw, Tim Shaw, Heather Shepherd, Rosalie Viney, Jackie Yim, Joanne Shaw

**Affiliations:** 1 The University of Sydney, School of Psychology, Psycho-Oncology Co-operative Research Group (PoCoG), Sydney, NSW 2006 Australia; 2 The University of Sydney, Faculty of Medicine and Health, Sydney, NSW Australia; 3 Chris O’Brien Lifehouse, Camperdown, NSW Australia; 4 Ingham Institute for Applied Medical Research, South Western Sydney Clinical School, University of New South Wales, Kensington, Australia

**Keywords:** Anxiety and depression, Cancer, Clinical pathway, Implementation, Staff perspectives

## Abstract

**Background:**

Clinical pathways (CPs) are intended to standardise and improve care but do not always produce positive outcomes, possibly because they were not adapted to suit the specific context in which they were enacted. This qualitative study aimed to explore staff perspectives of implementation of a CP for routine screening, assessment, referral and management of anxiety and depression (the ADAPT CP) for patients with cancer, focussing on perceived feasibility of the CP and negotiated adaptations made during the implementation phase.

**Methods:**

The ADAPT CP was implemented in 12 urban and regional oncology services in Australia. Services were randomised to receive core versus enhanced implementation strategies. Core sites received support until implementation commencement and could access progress reports. Enhanced sites received proactive, ongoing support during the 12-month implementation. Purposively selected staff were interviewed prior to implementation (*n* = 88) and 6 months later, half-way through the implementation period (*n* = 89). Monthly meetings with lead multi-disciplinary teams at the eight enhanced sites were recorded. Data were thematically analysed.

**Results:**

Six overarching themes were identified: ADAPT is of high value; timing for introducing the CP and screening is difficult; online screening is challenging; a burden too much; no-one to refer patients to; and micro-logistics are key. While early screening was deemed desirable, diverse barriers meant this was complex, with adaptations made to time and screening location. Online screening prompted by email, seen as time-saving and efficient, also proved unsuccessful in some services, with adaptations made to in-clinic or phone screening, or repeated email reminders. Staff negative attitudes to ADAPT, time constraints, and perceived poor fit of ADAPT to work roles and flows, all impacted implementation, with key tasks often devolving to a few key individuals. Nevertheless, services remained committed to the ADAPT CP, and worked hard to create, review and adapt strategies to address challenges to optimise success.

**Conclusions:**

This study demonstrates the interactive nature of health service change, with staff actively engaging with, forming views on, and problem-solving adaptations of the ADAPT CP to overcome barriers. Obtaining staff feedback is critical to ensure health service change is sustainable, meaningful and achieves its promise of improving patient outcomes.

**Trial registration:**

The study was registered prospectively with the ANZCTR on 22/3/2017. Trial ID ACTRN12617000411347.

**Supplementary Information:**

The online version contains supplementary material available at 10.1186/s12913-022-07532-2.

## Background

Clinical pathways (CPs) outline a sequence of standardised, evidence-based clinical management steps with timeframes and expected outcomes, for defined patient groups [1]. They are intended to be operational and detailed, to guide routine clinical practice. CPs have been shown to improve patient outcomes including mortality [2–4], increase hospital efficiency and decrease health service utilisation [5–9]. However, CPs in some studies have failed to demonstrate such benefits [10], possibly because of barriers experienced during implementation [11, 12] due to lack of fit to the specific context in which they were enacted.

Within the literature, there is significant debate regarding if, and to what extent, CPs should be adapted during implementation. Some advocate for strict adherence to the published CP [13, 14]; others argue adaptation to local circumstances and preferences is beneficial, provided stakeholder input guides a planned, systematic process [15, 16] and the core CP components are retained [17]. However, little is known about how stakeholders’ perspectives influence adaptations made to CPs, or the factors that influence those changes.

Our group developed a CP for screening, assessment, referral, and management of anxiety and depression in adult cancer patients (ADAPT CP) [18]. The evidence-based ADAPT CP was developed iteratively through a comprehensive stakeholder review and Delphi consensus process [19, 20]. It follows a stepped-care model incorporating screening at recommended intervals, triage to one of five steps, and referral for tailored treatment (from universal care and self-management for those with minimal anxiety and/or depression, to specialist care for severe cases), with review and change in step where necessary. Recommendations regarding staff roles, and content and timing of interventions, are provided for each step [18]. To facilitate CP implementation, we developed staff and patient education resources, and an online portal [21] to operationalise processes, increase efficiency and reduce staff time and burden.

We developed implementation strategies [22] to address identified barriers [19] to the ADAPT CP, including lack of ownership by staff, and poor fit for local resources, workflow and service culture. Strategies included allowing initial CP tailoring to local resources and preferences, and obtaining detailed feedback from staff at all levels prior to and mid-way through the 1-year ADAPT CP implementation period, to allow CP adaptation where possible in real time.

The ADAPT CP was implemented for 12 months, in a cluster randomised controlled trial (CRCT) evaluating two different implementation strategies (core versus enhanced) in 12 Oncology services in New South Wales (NSW) representing approximately 25% of cancer centres in the state, which were set in 7 of the 15 Local Health Districts of NSW, the most populous state in Australia [22]. The study was registered prospectively with the ANZCTR on 22/3/2017. Trial ID ACTRN12617000411347. The trial results will be reported elsewhere.

We have earlier described how the CP was tailored during the initial engagement with services prior to implementation [23]. This paper focuses on the perceived feasibility of the ADAPT CP during implementation, and changes required to accommodate experienced challenges, as part of planned reporting of implementation outcomes [24, 25].

## Methods

### Study design and setting

Participating sites were public or private oncology services providing cancer care for ≥ or < 100 patients per year (to examine how the ADAPT CP was used in sites with more or less patient throughput). Exclusion criteria included inability to commit to required study processes, and insufficient technology to support use of the ADAPT Portal. Potential sites (*n* = 12) were purposively selected to provide diversity in urban versus regional settings and size of patient load, and randomised to core or enhanced implementation support arms.

### ADAPT CP tailoring

At each service, a lead team comprising multidisciplinary leaders tailored the ADAPT CP to local requirements [23], a workflow was created, and the CP was implemented for 12 months [22] during which the lead team was responsible for discussing and resolving any issues arising. Two weeks after ADAPT CP implementation commenced, a start-up meeting was held with the lead team to discuss and resolve emerging issues (all services). Enhanced sites received proactive, ongoing support during the 12-month implementation, including monthly meetings with the ADAPT team to review portal activity reports and discuss any issues, ongoing awareness activities (staff posters and newsletters), additional meetings with the champion, and quarterly review of the portal for site-fit. Core sites received support until the start-up meeting and could access progress reports from the portal and initiate contact with the ADAPT team at any time but did not have scheduled meetings.

### Data collection

#### Staff interviews

Just prior to ADAPT CP implementation (T0), a subset of staff at each service, purposively selected to include members and non-members of the lead team and multi-disciplinary representation, were invited to participate in a semi-structured phone interview exploring attitudes to and expectations of the ADAPT CP. We aimed for a sample sufficient to ensure saturation of themes within different staff groups and different services.

Non-members of the lead team were included to ensure representation of views of frontline staff enacting the ADAPT CP. Interviews were conducted by three female researchers trained in qualitative methods with no involvement in preparing services to deliver the ADAPT CP. Interviews were conducted again 6 months later (T1) to obtain feedback regarding how the ADAPT CP was working in practice, any CP adaptations made and their rationale. The interview guide was developed based on a recent systematic review and implementation outcomes [12, 24] and was pilot tested. Data from T0 and T1 interviews were analysed for this paper. Interviews held at the end of implementation will be reported elsewhere.

#### Monthly meetings (enhanced arm)

Research staff minuted monthly meetings to capture discussion and decisions made.

### Analysis

Interviews were audio recorded, transcribed verbatim and coded in NVivo12 [26]. Data were thematically analysed to identify themes regarding adoption and feasibility of the ADAPT CP, adaptions made and their rationale. Two qualitative researchers, one who was involved in some interview data collection only, coded an initial six transcripts to develop a draft coding tree, which was discussed with a third researcher and refined. They then independently coded interviews line-by-line, with any differences resolved through consensus by the whole team. Similarities and differences in codes were examined to develop initial themes, which were reviewed to develop higher order themes. With the same coders and process, monthly meeting notes from the eight enhanced services were content analysed [27] and adaptations to local ADAPT CPs, including rationales, identified.

## Results

Seven metropolitan and five regional cancer services participated in the ADAPT CRCT (see Table 1). Publicly funded (*n* = 10), private (*n =* 1) and public-private partnership (*n =* 1) services were included.

**Table 1:** Service characteristics at commencement of implementation

Site ID	Site Location	Funding Type	Number of patients seen per 3-month period	Number of departments Included	Treatment modality departments Included	Tumour Streams Included	Number of streams included	FTE Psycho-social staff	Screening History in past 12 months
1	Major city	Public	≥100	3	Med Oncology Rad Oncology Haematology	All	≥3	0.8	Yes
2	Inner regional	Public	< 100	4	Med Oncology Rad Oncology Haematology Surgical	All	≥3	0.6	No
3	Inner regional	Public	< 100	1	Med Oncology	All	≥3	0.6	No
4	Major city	Public	≥100	2	Med Oncology Surgical	Gastro-intestinal	1	2.4	No
5	Inner regional	Public	< 100	3	Med Oncology Rad Oncology Haematology	All	≥3	1	Yes
6	Major city	Public	≥100	2	Med oncology Haematology	All	≥3	7.9	No
7	Major city	Public	≥100	1	Surgical	Upper GI	1	2.4	Yes
8	Major city	Public	< 100	3	Med Oncology Rad Oncology Haematology	All	≥3	5	Yes
9	Major city	Public	≥100	1	Haematology	Lymphoma, acute leukemia, multiple myeloma	≥3	2.4	No
10	Major city	Public	≥100	3	Med Oncology Rad Oncology Surgical	Head & Neck	1	4	No
11	Major city	Public and Private	≥100	1	Med Oncology	Sarcoma, Gynae	2	6.9	Yes
12	Major city	Private	≥100	1	Med Oncology	All	≥3	0.9	No

### Staff interviews

Of 126 staff invited, 88 consented and were interviewed at T0 (70% response rate) and 89 were interviewed at T1. Due to staff changes, leave or unavailability, different individual staff members were approached at T1; 64 staff were interviewed at both T0 and T1, 24 only at T0 and 25 only at T1. Average interview duration was 22 mins and 24 min at T0 and T1 respectively. Participants had on average 6 years of experience in their role (range 0 to 33). See Table 2 for participant demographics.

**Table 2:** Staff interviews: Demographic and professional characteristics

	T0 (***n =*** 88)		T1 (***n =*** 89)	
	n	%	n	%
Age Range (in years)				
18–25	2	2.3	2	2.2
26–50	61	69.3	67	75.3
51–75	23	26.1	16	18.0
Missing	2	2.3	4	4.5
Gender				
Female	75	85.2	73	82.0
Male	13	14.8	16	18.0
^a^Role				
Nursing Staff	33	37.5	34	38.2
Medical Staff	12	13.6	13	14.6
Allied Health and Clinical Trials Staff	6	6.8	4	4.5
Admin, technical support and non-clinical managers	15	17.0	12	13.5
Psycho-social Staff	22	25.0	26	29.2
Employment Status				
Full-time	57	64.8	58	65.2
Part-Time	27	30.7	26	29.2
Part-time, Independent Contractor	2	2.3	0	0.0
Full-time, independent contractor	0	0.0	1	1.1
Missing	2	2.3	4	4.5
Language spoken at home				
English	77	87.5	74	83.1
^b^Other	9	10.2	11	12.4
Missing	2	2.3	4	4.5
Country of birth				
Australia	62	70.5	58	65.2
^c^Other	24	27.2	27	30.3
Missing	2	2.3	4	4.5
Aboriginal or Torres Strait Islander				
No	85	96.6	84	94.4
Yes, Aboriginal	1	1.1	1	1.1
Missing	2	2.3	4	4.5

^a^Roles included in the categories:

Nursing Staff: Nurse- RN/AIN, CNS, CNE Care Coordinator, CNC, NUM, Nurse Practitioner

Medical Staff: Oncologist, Haematologist, Psychiatrist, Registrar, Medical oncology Fellow

Allied Health & Clinical Trials Staff: Speech pathologist, Clinical Trials,

Admin, technical support & non-clinical managers: Admin, IT staff, Volunteer, Clinical Support Officer, Management, Program Coordinator, Practice Manager

Psychosocial staff: Psychologist, Psychologist Intern, Social Worker, Counsellor

^b^Other languages spoken at home: Spanish, Mandarin, Cantonese, Indonesian, Portuguese, Tagalog, Malayalam

^c^Other countries of birth: UK, China, India, Indonesia, Brazil, Kenya, Hong Kong, Philippines, Sri Lanka, South Africa, Peru, New Zealand, Canada

### Monthly meetings

Across enhanced sites 84 monthly meetings were held *(M* = 10.50). Average meeting duration was 35 min. Forty-one meetings (49%) were delivered in-person, 35 (42%) online (web-conference), and eight (10%) via teleconference. There were 366 attendances across all meetings (*M* = 4.21 per meeting); the majority of attendees (*n* = 358, 98%) were lead team members (see Table 3).

**Table 3:** Monthly Meeting frequency, delivery mode, duration, and attendance

Site	Meeting Mode and Frequency				Meeting Duration		Meeting Attendance		
	Number of In-person Meetings (n)	Number of Online Meetings (n)	Number of Tele-conference Meetings (n)	Total Number of Monthly Meetings (n)	Total Meeting Duration (mins)	Average Meeting Duration (mins)*	Lead Team Attendances (n)	Non-Lead Team Attendances (n)	Total Attendances (n)
Site 2	2	5	3	10	331	33	59	0	59
Site 4	2	7	1	10	400	40	54	0	54
Site 5	0	11	0	11	310	28	33	0	33
Site 6	7	3	1	11	545	50	46	4	50
Site 7	10	0	0	10	310	31	33	0	33
Site 8	3	9	0	12	330	28	40	2	42
Site 9	10	0	0	10	320	32	56	0	56
Site 10	7	0	3	10	300	30	37	2	39
Total	41 (49%)	35 (42%)	8 (10%)	84 (100%)	2846 (47 h 26 min)	34 mins	358 (98%)	8 (2%)	366 (100%)

*Average meeting duration = Total meeting duration (mins)/ number of monthly meetings (n) for each service

### Themes

Qualitative analyses identified six overarching themes: ADAPT CP is of high value; timing for introducing the CP and screening is difficult; online screening is challenging; a burden too much; no-one to refer patients to; and micro-logistics are key. Role, site (S) participant ID (P), and assessment point (T) are identified for each interview quote e.g., NURS_S01P05T1. See Table 2 for included professions. Data from monthly meetings are identified as MM. Additional quotes to support each theme are provided in Table 4.

**Table 4:** Additional quotes illustrating themes

Theme	Supporting quotes
ADAPT is of value	*“Yeah. I think, I think staff generally, … understand the value of it and, and are accepting of it. Definitely*.” (ADMIN_S08P05T0) *“It’s wonderful. When I heard about it, I thought great - finally, we’re going to get some direction here. And that’s what’s happening.”* (NURS_S02P04T0) *“There is just more recognition that this is important for patient care. And that, um, anxiety and distress are important aspects, um, to address and so I think there is universal recognition that we need to do this*.” (MED_S01P09T1) *“[Psychosocial staff, Champion] said it does catch some missed people which is useful considering how busy psychology is.”* (S6, MM) *“[ADAPT Program Manager] asked team to consider how well ADAPT is working on a 1–10 scale. [Psychosocial staff] rated it an 8 as it brings people to her attention that she may not have otherwise seen or picked up.*” (S5, MM)
Timing was a challenge	*“if … they’re told about it so early on in the … treatment and there are so many other things going on, … it’s probably like yep, yep, yep … but at the same time they don’t really process this, and it doesn’t really matter six weeks or whatever it is later”* (PSYCH_S08P04T1) *“The team commented that in their service, introducing ADAPT to patients is difficult because there are multiple entry points within the service”* (S8, MM)
Online screening fails as a solution	*“I think, having the system online or* via *the phone … I think, is another barrier. I just think it’s so much easier to brush off so not – not do your screening or what not. If you’re not, kind of, forced to”* (NURS_S07P06T1)
A burden too much	“*I think other staff are receptive. I just think it’s time, people being time poor, all of the time*” (NURS_S09P08T1). “*… with competing demands here of inpatient work, and crisis in the clinic, it’s been really hard to get those screened ones done as timely as I would like and I’m hearing that’s the same from the care coordinators.”* (PSYCH_S01P03T1) *“It’s not what we specifically trained for in terms of clinical psychology therapy … it’s not the best use of our time I don’t think, and it’s probably not the best use of hospital money to give someone the distress monitor all day”* (PSYCH_S06P05T1) *“… the team discussed issues and barriers to patient registration. [Psychosocial Staff, Champion] feels that relying on doctors to recognise that a patient meets the ADAPT criteria is not realistic.”* (S4, MM). “*… whilst there is support in principle for screening from medical staff there is not scope for medical staff to take on any role in introducing this approach to patients …. [Psychosocial staff, Champion] noted he explained that this could be as little as 45 s to 1 min, but this was still considered too much.*” (S9, MM) *“… If people are doing their screening while they’re in the oncology unit … and the nursing staff don’t have time to do it, then they’ll call me to come and help … set if up for the person, so that it gets done”* (PSYCH_S02P01T1) “*I’m really lucky in – in that I have a lot of support from the trial staff …. So that worked – that worked well for me*.” (NURS_S03P04T1)
No-one to refer to	“*So we don’t have a psychologist on site, um, and getting – for patients to get a referral to a psychologist is quite a, um, it can be quite a difficult, um, process, um, because to get to a psychologist you need mental health plan, but to get a mental health plan you need a GP appointment. Sometimes that can take three to four weeks to get a GP appointment.”* (AH_S03P06T0) *“… Um, I think that at where I work currently that there is lack of, um, services available, that, you know, we don’t have a psychologist full time and that a patient cannot just drop in and often there’s a large waiting time*.” (NURS_S04P03T0)
Micro-logistics	*“the suggestions were made to utilise the Advanced Trainee ([Medical staff]‘s patients only) and also to have the pamphlet and registration slip attached the front of new patient folders so that doctors see it.”* (S5, MM)

#### 1. ADAPT CP is of high value

Attitudes towards ADAPT CP were positive throughout implementation. Participants believed the ADAPT CP would ensure all patients who needed psychosocial help would be identified, including those with non-visible distress, facilitate early identification of morbidity to enable timely and appropriate care, and keep patients positively connected to the service.

*“I think it [ADAPT CP] is needed and useful, I think everyone knows it’s common for cancer patients to have anxiety and depression, we all recognise that treatment is useful*.” (MED_S11P05T0).

*“It’s seen as a … positive way to stay engaged with the team even after discharge*.” (PSYCH_ S07P05T0).

*“I’ve had a couple of patients actually thank me about it, … just reaching out … I think … they’ve found that quite nice*.” (NURS_S01P04T1).

*“The staff have said that, because of ADAPT, we have picked up patients who needed help even though they didn’t appear to need help.*” (MED_S01P09T1).

However, some felt ADAPT was *“somewhat redundant”* (S8, MM) and duplicated existing services, “*re-inventing the wheel*” (PSYCH_S12P07T1).

*“To be honest, I think we had a pretty good way of doing it before ADAPT … but it’s good to have a bigger version and it’s online so people can do it self-directed”* (NURS_S07P04T0).

#### 2. Timing for introducing the CP and screening is difficult

Despite positive attitudes, challenges to implementing ADAPT CP were identified by staff, including timing. Some sites initially chose to introduce ADAPT and the rationale for regular screening at the patient’s first oncologist appointment. However, patients were sometimes hard to identify at this time (due to multiple entry points into the system) and too overwhelmed. Staff were focused on co-ordinating treatment plans and yet to develop a relationship with the patient. If some time elapsed before the patient was screened, some patients forgot or lost motivation.

*“I think it’s given at the wrong time … they have a thousand things to think about when they’re first seeing the doctors in that situation. So … I think that’s very inappropriate”* (NURS_S04P03T1**).**

*“Team says if the patient does not know them at this stage and if it is introduced over the phone, it could be difficult to sell.”* (S2, MM).

Screening in clinic immediately prior to or during treatment also presented issues, as anxiety and depression scores could be transiently and contextually high. Staff also found introducing ADAPT during clinics was “*tough*” for nursing staff on top of existing clinical responsibilities, and time consuming.

“*… we had a few people that have screened while they’re having treatment, … they can screen quite high because they’re quite anxious just about how it’s going to go, and what’s happening and it’s all new... Whereas if they did it at home or after their treatment, then they might actually have screened differently.”* (PSYCH_S02P01T1).

##### 2.1 Adaptations to address timing difficulties

After discussion with the ADAPT team, several services changed to later registration which was perceived to work better:

*“We were doing the distress thermometer on first presentation and so, there was an awful lot of people that I was contacting. So now … they’re screened more appropriately and at a better time. because everyone’s going to flag high on initial presentation … it’s culled a bit of work out.”* (PSYCH_ S05P01T1).

One service shifted from introducing ADAPT at the surgical clinic to discharge planning, although even this was not deemed optimal:

*“[Nursing staff, Champion] emailed staff regards inclusion of ADAPT in discharge planning. She feels this has had no impact on ADAPT activity, mainly because patients are very pre-occupied with other issues at discharge.”* (S7, MM).

One nurse noted that conducting screening in the chemotherapy suite was “*more feasible*” as patients were there much longer (PSYCH_S03P08T1). Another said:

*“So I’ve spoken to all my colleagues … we feel to introduce them to the ADAPT program is best when the nurse does drug education … and then when they come in to Cycle 1 in the chair … we then say, would you like to register? And if they do, we register there and then.”* (NURS_S02P09T1).

One site organised a back-up approach by the Nursing Unit Manager if ADAPT was not introduced; other sites simply accepted that some patients would be missed.

#### 3. Online screening is challenging

While initially thought to offer considerable benefit in saving time and providing greater convenience to patients, online screening prompted by email had a low uptake, and even when patient-preferred, low adherence (only 57% of patients choosing email actually screened). Staff cited barriers such as older age and lower socio-economic background contributing to patients not having an email address, lacking access to devices at home or lacking the technological literacy to complete email screening independently. Furthermore, without staff present to prompt screening, it was quickly forgotten by patients.

*“We’d imagined that people would register and then they would screen via emails and electronically, but that hasn’t happened.”* (PSYCH_S10P01T1).

*“We have quite an older population here, so a lot of people don’t have email addresses.”* (AH_S06P03T1).

*“A few people have said, oh, yeah, yeah, I remember that being in my inbox and, um, they just don’t want to address it really.”*(NURS_S12P01T1).

However, some staff believed that the way ADAPT was communicated to patients was critical in influencing adherence, as opposed to the medium or format of screening.

*“I think … that we have to push it so that it’s almost not optional … this is what we do; how do you want to receive this? Do you want to do it here, or do you want to do it at home?”* (NURS_S02P08T1).

One site identified two instances where family members had completed email screening without patients being aware this had taken place (S6, MM), which raised concerns. Another site became concerned that email screening may lead to delays in offering support to patients expressing high distress, violating their duty of care.

*“[Nursing staff] was questioned … about patients screening highly distressed/suicidal on a Friday night and then this not being acted on until a Monday and conveyed it would not be acceptable to leave them 3 days without contact.”* (S2, MM).

##### 3.1 Adaptations to address online screening challenges

In the case above, it was ultimately decided that since patients are automatically referred by the online portal to resources and helplines if their distress score is high, and since a direct suicide question is not asked, then online screening by email could continue. One site developed an email reminder system for patients if they did not screen immediately:

*“Team reconfirmed that email is preferred as finding people in clinic and getting hold of them over the phone can be logistically difficult and time-consuming … Email reminders sent by [Psychosocial staff, Champion] to remind patients to screen.”* (S6, MM).

Other sites reverted to in-clinic screening or telephone follow-ups with patients to prompt screening.

#### 4. A burden too much

Despite initially reporting that the ADAPT CP fulfilled service and personal mission, many staff felt ADAPT tasks were “*not my role*,” that they lacked required skills, or just could not fit them into their already stretched workload, and that ADAPT caused friction. A social worker said:

*“Nurses are run off their feet … Nurses cannot and will not prioritise this work”.* (S6, MM).

*“They’re like, that’s not my thing, that’s not my job …*” (NURS_S01P06T1).

*“I also get a sense that there might be some hesitation to actually discuss anxiety and depression … they [nursing staff] are worried that if they open a can of worms, the patient will … almost decompensate, and what do they do?”* (PSYCH_S06P13T1).

In one site, the view was expressed that ADAPT would never be accepted unless it was made mandatory:

*“[Psychosocial staff, Champion] noted that the only way people will engage with it is if this is mandated …*. *if [Med Onc] and [Director of Cancer Services] make them do it.”* (S10, MM).

Conversely, other staff felt that ADAPT had reduced their burden.

“*If anything, it’s probably made things a little bit easier for us, [because] we can pick the patients up earlier …. rather than waiting until things fall down and then, we’re trying to pick them back up*.” (NURS_S01P04T1).

##### 4.1 Adaptations to address staff burden challenges

Some services put significant thought into role responsibilities to maximise efficiency and efficacy, and make use of existing staff skills, both initially, and over time.

*“We’ve got the girls out the front. We’ve got it set up that they’ll be able to help them out once … the patient has been educated about the program and the screening …”* (ADMIN_S02P05T0).

*“We were mindful during the planning stage of … who’d have the clinical confidence to go asking questions or running through surveys with patients, so admin were delegated tasks which were specific to their role, and nursing staff and the new [psychosocial staff] were allocated roles which were according to their competencies.”* (NURS_S05P06T0).

Some sites tried to reduce burden by involving more staff to share the load.

*“ADAPT is ‘everybody’s business’ such that all people need to be involved rather than this being the responsibility of a single person”* (S8, MM).

*"… there’s five of us. So we each just take one day and then if … an alert comes on in that day then it’s our responsibility to do the triage conversation”* (PSYCH_S02P01T1).

In some sites, however, back-up staff were unwilling to help out, particularly to pick up patients not seen by the designated screener. In other sites, roles devolved to only a few, often the champion or lead team, to ensure tasks were completed.

*"This theme has been raised consistently throughout the implementation, but attempts to engage medical staff, ward staff, step down facility staff, outpatient staff have failed.”* (S9, MM).

*“So we have a lead team … we were meant to share the workload between us …. But it’s ended up being … just between three of us … and I think that’s what’s been so difficult”* (NURS_S10P02T1).

However, restricting ADAPT roles to the lead team could mean that most clinical staff lost awareness of ADAPT, which became *“invisible on the ward.”*

*“They’re heavily involved in it and they’re managing it quite well. So it’s … almost to the point, non-existent for everyone else on the ward.”* (NURS_S07P06T1).

Finally, if the person responsible for screening went on leave, or was sick, the system broke down:

*“Registrations: have been lower than expected over the last 2 months and it became apparent that in the absence of the administrator who had been placing pamphlets and registration slips, this task had not been completed by anyone else which led to a breakdown in the workflow.”* (S6, MM).

One site decided to make adaptations by devolving some of the work to patients themselves, by prompting patients to screen via email and complete their own registration:

*“If all patients screen via email and patients fill out mandatory fields themselves, it could be possible to eliminate the registration slip step...”* (S4, MM).

#### 5. Nowhere to refer patients to

Some services had no, or insufficient, inhouse psychosocial staff, and were concerned that having identified psychological morbidity, they could not facilitate appropriate and timely support. Some services relied on referral to a local GP, private providers or a psychiatrist.

*“… at the moment, … we have only got one clinical psychologist … and so, that is quite difficult … for our patients to get into that, so … some people that potentially could, afford to … pay to go to see somebody privately...”*(NURS_ S01P04T0).

*“We don’t have much psychosocial support here at all. We have a social worker.”* (NURS_S03P05T0).

There was also a perception by staff that external pathways were not always ideal or were difficult for patients to access. However, this was the only option for referral at some services in the absence of inhouse psychosocial staff.

##### 5.1 Adaptations to address referral challenges

Some services saw this as an opportunity and took the initiative to expand referral sources when gaps were identified in planning to implement the ADAPT CP.

*“Pathways for these patients to go to, apart from the social worker … are starting to be developed which is great”* (NURS_S03P04T1).

*“We’ve got access now to a Telehealth interview with an area psychiatrist who is providing psychology services one afternoon a month … for those higher-level distress patients. It’s better than it has been, because … [previously] we have to contact the GP and ask them to do a mental health plan. Then they get put on a waiting list for, um, to see a psychologist in the community. And that’s still under-resourced and hasn’t changed.”*(NURS_S02P08T1).

#### 6. Micro-logistics are key

Some services discussed workflow breakdowns due to clinic processes that did not emerge until implementation was underway, for example, unclear procedures or inadequate record-keeping.

##### 6.1 Adaptations to address micro-logistics

Many services innovatively developed their own systems for ADAPT CP processes, such as who did what, placing ADAPT registration forms in patient files, providing a script for staff to use when introducing ADAPT, and having an agreed sequence for paperwork so that missing steps could easily be identified and rectified. These adaptations were well received.

*"So we got the flow sheet … and tweaked it and … even during training as other things came up … with the volunteers and their concerns, things have been tweaked again … which has been really good … that has been able to work for us."* (NURS_S01P04T0).

*"So … they’ve come up with a whole practice around that which they’ve highlighted and mapped out and done that very well so far, so, happy with the way they’re working on those."* (ADMIN_S01P10T0).

*"Suggestion by [CNC, Portal Champion] was the New Patient Form have an ADAPT tick-box included so specialists remember to raise it with patients"* (S4, MM).

Staff noted that the initial thought that went into developing systems could make or break the success of the CP.

*“And I do think all those little things add up … just make it easier when you’re in the clinic and you’re trying to think of ten things, and this one just can tell you automatically”* (MED_S06P02T1).

### Formal changes made to the ADAPT CP workflows

While many services adapted the ADAPT CP, services varied in the degree to which they formally notified ADAPT research staff of changes leading to the local workflow being updated. Five services notified no changes, four 1 change and three 2 changes. Examples of initial and final detailed service workflows for two services are provided in Additional file 1. Overall, however, ongoing thought and commitment was evident in most services, with adaptations occurring throughout to support adoption and feasibility of the implementation.

## Discussion

This is one of the first studies to document stakeholder experiences of implementing a CP, and the adaptations to the CP made as a result to address feasibility issues. The CP in this case was for screening and management of anxiety and depression in cancer patients (the ADAPT CP) [18].

Despite steps taken to maximise the ADAPT CP implementation success (e.g., designing the CP and its implementation in response to pre-identified barriers, and tailoring the CP to local resources and requirements prior to implementation), and a belief expressed by many stakeholders in the value of the CP, barriers to its adoption and implementation feasibility were still encountered.

These included finding the right time to initiate screening when patients had formed a trusting relationship with staff, were no longer overwhelmed, yet were sufficiently early in their care to allow identification of distress before it developed into a clinically significant problem. The Australian Clinical Practice Guidelines for managing anxiety and depression in cancer patients [18] do not specify timing of the initial screen, but they do promote early identification of distress, whereas the US National Cancer Control Network (NCCN) recommends all patients are screened for distress at their initial visit, and subsequently at appropriate intervals or as clinically indicated [28]. A clear evidence-base documenting the quality of life and economic impact of early versus later screening is lacking, thus there is little guidance for services grappling with this decision. Our experience suggests the optimal timing (and location) for initial screening will likely depend on service workflow; staff may need to seek repeated opportunities to ensure patients are not missed.

A second issue which emerged was the virtue or otherwise of online screening. Online screening has been promoted as a way to reduce costs and increase feasibility and acceptability for patients who may be too rushed or uncomfortable to complete questionnaires in the clinic [29], and decrease time and resources required for staff to supervise screening [30]. However, reported disadvantages include that patients who are older or less computer literate may be less likely to participate, decreasing response and retention rates [31]. This barrier may change over time, but for now, requires some flexibility and tailoring to patient demographics and preference.

Another key barrier to implementation of the ADAPT CP identified by participants, was the perceived staff burden, on top of existing roles and responsibilities, of required tasks related to ADAPT. The degree to which this activity was felt to be a reasonable and valid part of diverse staff’s roles, core to service and individual mission, impacted the way services responded. As Damschroder et al. [32] has commented, the overarching culture and climate of workplaces, often neither explicit nor acknowledged, are key to achieving practice change. An open discourse within services about mission and expectations regarding patient-centred care, support from management for activities in this arena, and collaborative and integrated systems for delivering care, are all likely to support staff to work together to deliver psychosocial care, as has been argued elsewhere [33].

Management could also choose to strongly endorse participation in new psychosocial CPs and perform audits to ensure compliance. Furthermore, at a service level, adequate resourcing is vital to allow staff to complete required tasks without risking stress and burnout. A recent Australia survey found 38% of respondents reported lack of resources as a major barrier to implementing psychosocial care [34]. Despite wide-spread acknowledgement of the importance of context and facilitation to implementation success (such as within the Promoting Action on Research Implementation in Health Sciences (PARiHS) framework [35]), health systems often fail to commit adequately to service change and provide sufficient resourcing [36]. This is particularly true of mental health initiatives. For example, in England, the Health and Social Care Act 2012 established parity for mental and physical health, yet while mental health accounts for 28% of the disease burden, it attracts only 13% of NHS spending [37]. Where possible, services need to argue for adequate resources, supported by strong health economic data, *prior* to implementing a new CP.

In the current study, many services made creative adaptations to overcome challenges, demonstrating the key importance of leadership and active engagement by a range of stakeholders (delivery partners) in implementing change, as noted by Yamey et al. [38]. Without the contribution of staff who know the intricacies of the health system, and the support of management, health system change is bound to fail. The small micro-logistic changes made by our participating services to fit with work and patient flow sometimes made the difference between success and failure. They are an under-recognised contributor to implementation success that deserve larger recognition in implementation frameworks and guidelines.

This study is not without limitations. While we had good multidisciplinary representation, some staff did not agree to be interviewed, thus their views were not represented. While interviewers were independent of the ADAPT research team who interacted with staff during implementation, social desirability bias may nonetheless have impacted responses, leading to a more positive perspective being presented than was actually felt. Due to more intensive interaction of ADAPT Team with Enhanced arm sites during implementation (e.g., during monthly meetings), there may have been barriers/challenges and adaptations at Core services during the implementation phase to which the ADAPT Staff were not privy and thus not reported here.

## Conclusions

As noted by Simmons et al. [39] *“Most good ideas … do not spread with such ease. They require the backing and energies of committed individuals and organizations to design and carry out strategies for expansion that are carefully tailored to the realities of their settings.”* Chambers and Norton [40] have argued it is essential to design interventions that build on existing processes and capture local knowledge to ensure local fit *before* they are widely implemented. Furthermore, they argue it is important to recognise that interventions are adapted all the time. It is therefore critical that real world adaptation is not ignored, but rather documented and studied so that we can better understand how and why some interventions are more sustainable than others. In this study, we documented staff perspectives just before and midway through implementation of a clinical pathway to identify and manage anxiety and depression in patients with cancer, and the adaptations made to the CP to overcome feasibility and adoption challenges, adding to the scarce literature on intervention implementation in real-world studies.

## Supplementary Information

**Additional file 1.** First and final ADAPT workflows for two contrasting sites. Red indicates where steps have been added to the workflow, blue indicates where adaptations to previous workflow decisions have been made.

## Data Availability

Data and materials are available from the Corresponding author on request.

## Abbreviations

CP: Clinical Pathways
ADAPT CP: Clinical Pathway for the Screening, Assessment and Management of Anxiety and Depression in Adult Cancer Patients: Australian Guidelines
ADAPT: The Anxiety and Depression Pathway Program. A Translational Program Grant: A Sustainable and supported clinical pathway for managing anxiety and depression in cancer patients.
ADAPT Portal: Web-based system to operationalise the Clinical Pathway for the Screening, Assessment and Management of Anxiety and Depression in Adult Cancer Patients: Australian Guidelines
CRCT: Cluster Randomised Controlled Trial
