# Supplementary material for: Staff perspectives on the feasibility of a clinical pathway for anxiety and depression in cancer care, and mid-implementation adaptations
Source: BMC Health Serv Res. 2022 Feb 14;22:192. doi: 10.1186/s12913-022-07532-2 (PMC8842573; doi:10.1186/s12913-022-07532-2)

Additional File 1. First and final ADAPT workflows for two contrasting sites. Red indicates where steps have been added to the workflow, blue indicates where adaptations to previous workflow decisions have been made.

Site 1. Workflow a (at Go-Live)

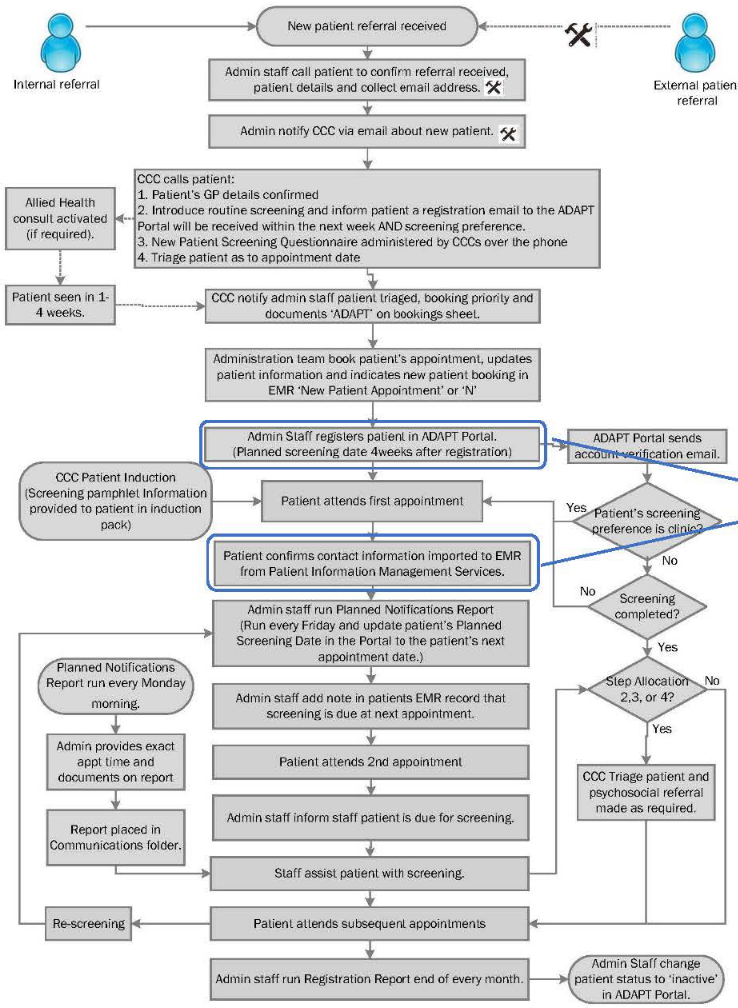

Site 1. Workflow b

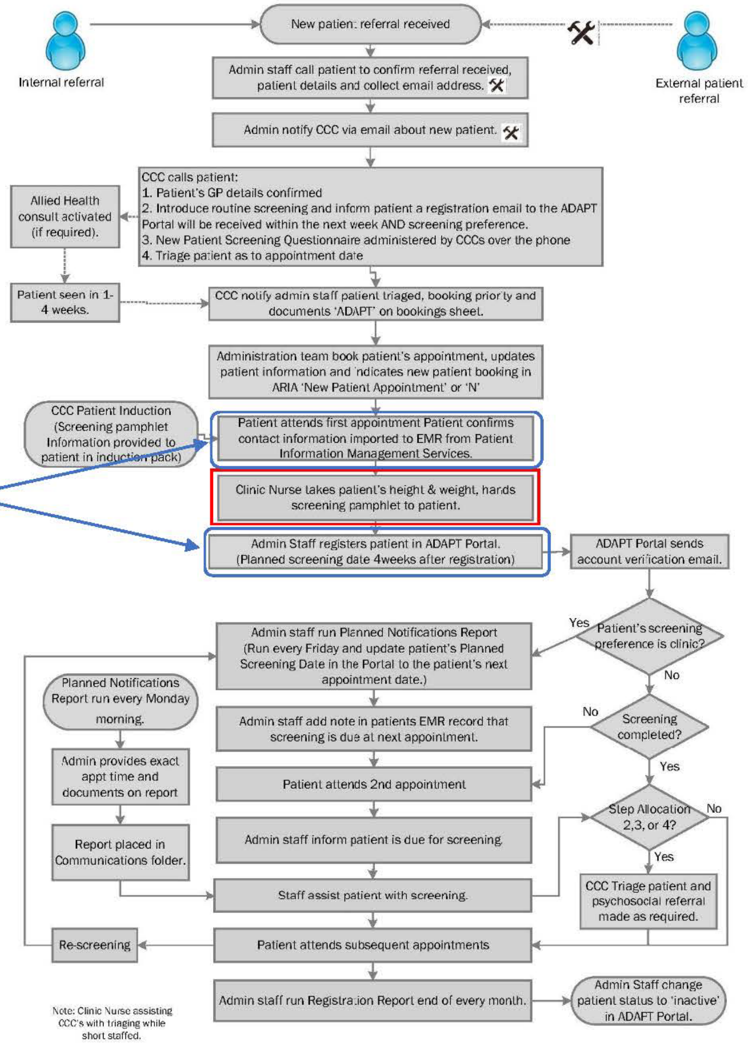

Site 6. Workflow A (at Go-Live)

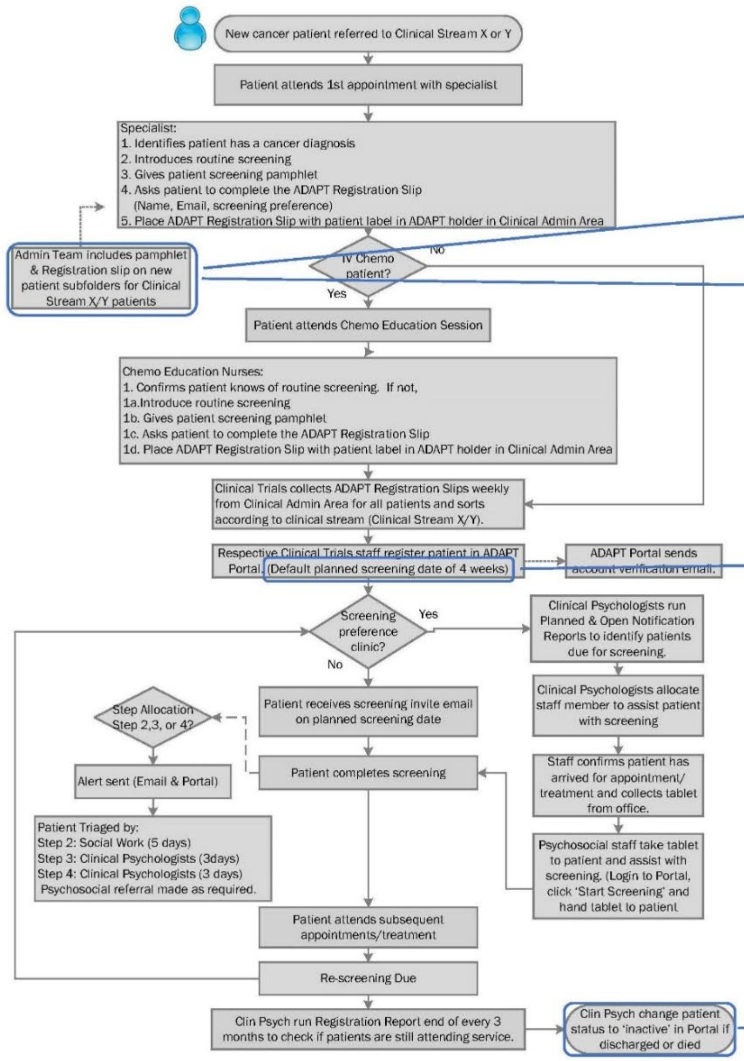

Site 6. Workflow B

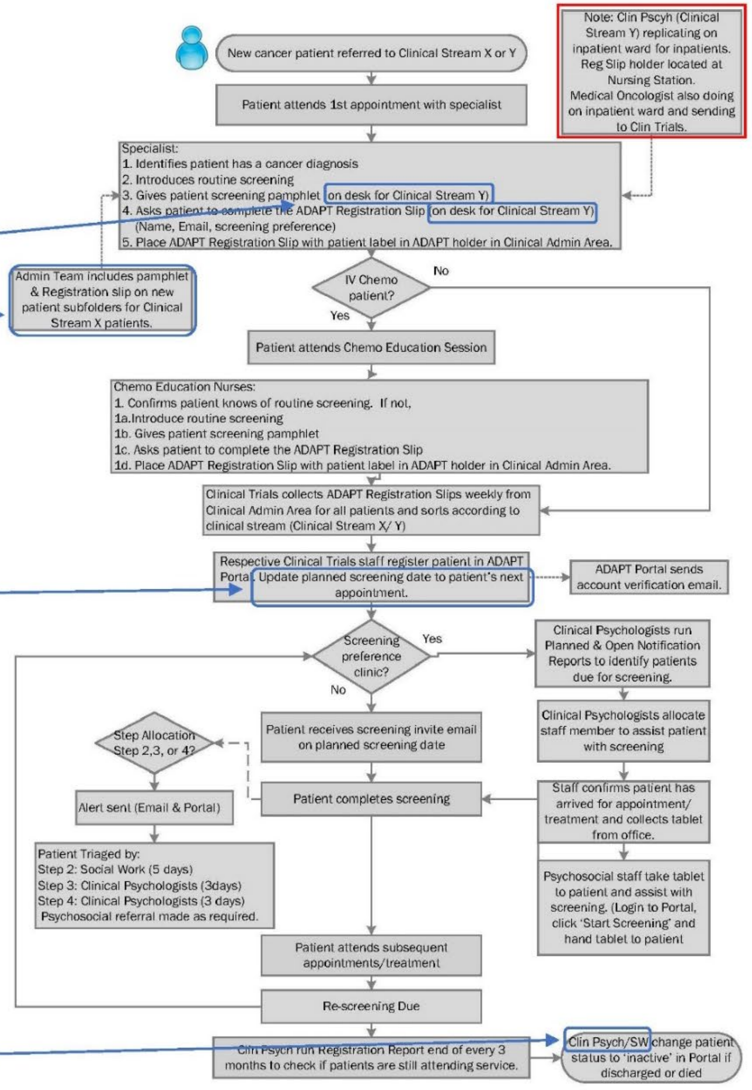

Supplement: Supplementary file 1 — Additional file 1. First and final ADAPT workflows for two contrasting sites. Red indicates where steps have been added to the workflow, blue indicates where adaptations to previous workflow decisions have been made. [file 12913_2022_7532_MOESM1_ESM.pdf]
